# Antibacterial Activity against Methicillin-Resistant *Staphylococcus aureus* of Colloidal Polydopamine Prepared by Carbon Dot Stimulated Polymerization of Dopamine

**DOI:** 10.3390/nano9121731

**Published:** 2019-12-04

**Authors:** Moorthy Maruthapandi, Michal Natan, Gila Jacobi, Ehud Banin, John H. T. Luong, Aharon Gedanken

**Affiliations:** 1 Department of Chemistry, Institute for Nanotechnology and Advanced Materials, Bar-Ilan University, Ramat-Gan 52900, Israel; lewismartin.jesus@gmail.com; 2 The Mina and Everard Goodman Faculty of Life Sciences, Institute for Nanotechnology and Advanced Materials, Bar-Ilan University, Ramat-Gan 52900, Israel; natan.michal@gmail.com (M.N.); gilajacobi@gmail.com (G.J.); banine@mail.biu.ac.il (E.B.); 3 School of Chemistry, University College Cork, Cork T12 YN60, Ireland; luongprof@gmail.com

**Keywords:** carbon dots, polydopamine, colloidal dispersion, *Staphylococcus aureus*, eradication of MRSA

## Abstract

A simple one-step process for the polymerization of dopamine has been developed using nitrogen-doped carbon dots (N@C–dots) as the sole initiator. The synthesized amorphous polydopamine (PDA)-doped N@C–dots (PDA–N@C–dots composite) exhibited a negative charge of –39 mV with particle sizes ranging from 200 to 1700 nm. The stable colloidal solution was active against methicillin-resistant *Staphylococcus aureus* (MRSA), a Gram-negative bacterium. The strong adhesion of the polymer to the bacterial membrane resulted in a limited diffusion of nutrients and wastes in and out of the cell cytosol, which is a generic mechanism to trigger cell death. Another possible route is the autoxidation of the catechol moiety of PDA to form quinone and release reactive oxygen species (ROS) such as superoxide radicle and hydrogen peroxide, two well-known ROS with antimicrobial properties against both Gram-negative and Gram-positive bacteria.

## 1. Introduction

Carbon quantum dots (QDs) or simply carbon dots (CDs) exhibit excellent biocompatibility, low cytotoxicity, high chemical stability, optical transparencies, conductivity, high mechanical strength, and narrow bandgaps [1,2,3,4,5,6,7]. Of interest are N-doped CDs with a high fluorescence quantum yield (44%) [8,9,10,11,12,13,14,15,16,17,18,19] as novel materials for diversified applications in sensing, electrocatalysis, solar cells, etc. Electron-accepting abilities of photoluminescent CDs stimulate chain transfer polymerization and free radical polymerization of water-soluble vinyl monomers [20]. Other reports unravel the use of CDs as co-initiators and catalysts. Some examples include free radical frontal polymerization, controlled light-mediated radical polymerization, and co-catalyzed polymerization [20,21,22,23]. Polymers with amino functional groups, such as polyaniline, polypyrrole, and poly (4,4′-diaminodiphenyl methane), were synthesized by CDs under UV light as an initiator [24,25]. Two polyether polymers, poly-(4,4′-oxybisbenzenamine) and copolymers involving the poly-(4,4′-oxybisbenzenamine), were synthesized by CDs in the absence of UV light [26]. To our knowledge, the use of N-doped CDs for polymerization processes has not been attempted.

Dopamine (DA; 4-(2-aminoethyl) benzene-1,2-diol) plays various physiological roles in mammals including neurotransmission. The lack of this chemical in the brain is attributed to the progressive neurological ailment known as Parkinson’s disease. Of notice is the remarkable adhesiveness of polydopamine (PDA), which has inspired biomedical applications such as coatings for cell interfacing, drug delivery, and biosensing. Although most polymers exhibit some degree of adhesiveness, this property is predominant in PDA [27], which carries amino, imino, hydroxyl, and catechol functional groups. π–π interactions are also anticipated with the host considering the aromatic ring of the PDA polymer. In this context, PDA can form a stable film on both inorganic and organic material surfaces with enhanced hydrophobicity. Another important feature of PDA is its antimicrobial property, as PDA binds a cell membrane to form a barrier on a cell surface to prevent the diffusion of nutrients and wastes in or out of the cell cytosol, leading to cell lysis. PDA is an important polymer with excellent biocompatibility and low cytotoxicity, which can be used for various applications [28,29,30] including biological/medicinal applications [31,32]. The PDA composites, particularly those decorated with metal oxides, have been used for cancer therapy and antibacterial treatments. These composites include PDA@SiO_2_ [30], Fe_3_O_4_@PDA [31], GO@PDA@GNS [32], AuNPs@PDA [33], WO_3_@PDA–HA [34], Mn_3_O_4_@PDA [35], Ag@PDA/graphene oxide sheet [36], PDA@Ag [37], MoS_2_–PDA–Ag composite [38], Ag–PDA–TiO_2_ composite [39], and PDA-coated sericin/polyvinyl alcohol [40]. Nevertheless, the preparation of such polymer-metal/metal oxide composites requires long synthesis time and expensive or toxic materials. It is also of high desire to develop a fast and simple method for the fabrication of PDA and PDA nanoparticle-based materials for various promising applications. Worth noting is the use of Fe^2+^/H_2_O_2_ to generate reactive oxygen species (ROS) for the accelerated self-oxidative polymerization of DA [41]. The study describes a simple and green method to trigger DA polymerization using only nitrogen-doped carbon dots (N@CDs) as an initiator. The resulting colloidal material was stable and then evaluated against methicillin-resistant *Staphylococcus aureus* (MRSA) as a test model. Moreover, at a minimal concentration of the PDA–N@CDs composite, a distinct and very high synergetic effect of the colloidal material was observed. As the most dangerous bacterium among all common staphylococcal bacteria, this Gram-positive and sphere-shaped (cocci) bacterium often causes skin infections that can be attributed to pneumonia, heart valve infections, and bone infections. This pathogen secretes toxins including potent hemolysins and leukotoxins to damage the biological membranes of host tissues.

## 2. Materials and Methods

Bovine serum albumin (BSA), NaOH, and DA were purchased from Sigma–Aldrich.

### 2.1. Synthesis of N@CDs

N@CDs were synthesized hydrothermally by dissolving 0.4 g BSA (66.5 kD) in 60 mL of doubly distilled water (DDW), followed by the addition of 10 mL of 0.1 M NaOH with stirring for 10 min at room temperature. This solution was transferred to a 25 mL Teflon-lined autoclave and heated at 180 °C for 6 h in a hot air oven. The supernatant solution was collected by centrifugation at 8000 rpm for 20 min. The resulting yellow solution was then subject to characterization.

### 2.2. Synthesis of Polydopamine by Homogenization

**Scheme 1:**
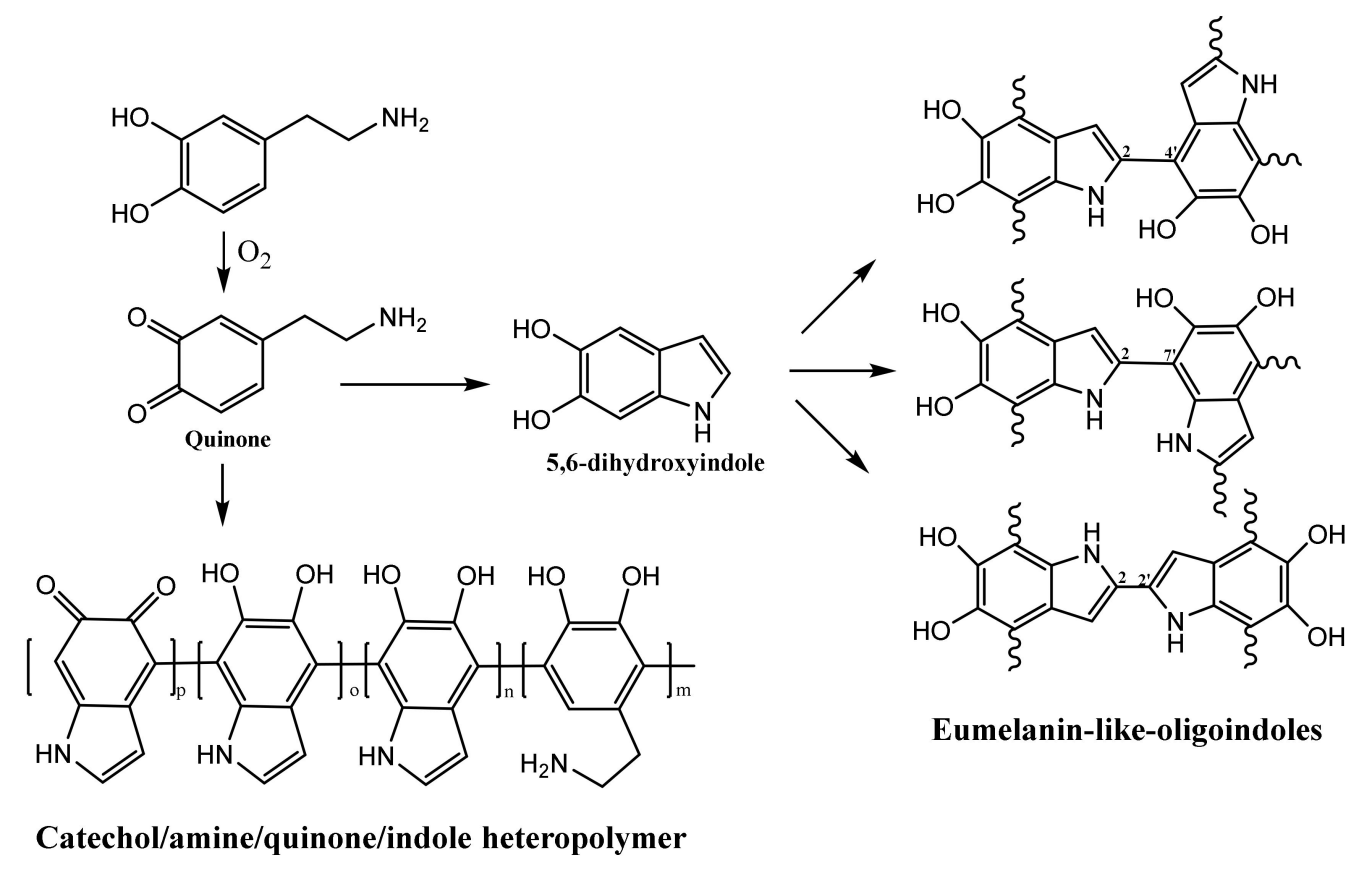
The plausible formation of various analogs of polydopamine (PDA) from dopamine (DA) by nitrogen-doped carbon dots (N@CDs). Redrawn from [42].

An aqueous solution of the N@CDs (40 mL with 0.25 g of the CDs) and 40 mL of distilled water were placed in a 100 mL beaker together with 0.5 g of DA. The solution kept in a homogenizer (its speed was 3000 rpm with a maximum speed of 30,000 rpm) at room temperature changed its color from yellow to dark brown after 20 min. After 5 h, the black solid was precipitated, indicating the complete formation of the polymer. The black solid was then collected by centrifugation at 10,000 rpm for 30 min, washed several times with distilled water, and dried at room temperature. The colloidal solution of the PDA–N@CD composites was removed before collecting the black precipitate from the reaction mixture. The colloidal solution of the PDA–N@CD composites was used to test its antibacterial activity. The polymerization of DA to form PDA was based on current theories stemming from the autoxidation of DA, leading to the formation of DA quinone and 5,6-dihydroxyindole [42]. The covalent coupling of these two intermediates formed either a catecholamine/quinone/indole heteropolymer or eumelanin-like oligoindoles (Scheme 1). The exact structure of the PDA–N@CD composite is not known, and this is a subject for future endeavors.

### 2.3. Analysis and Characterization

For high-resolution scanning electron microscopy (HRSEM) imaging on an FEI Megallon 400L microscope, a small amount of the dried polymer powder was placed on a carbon tape attached to a copper plate, followed by sputtering a thin gold film on the powder. The working distance was 6–7.1 mm, and the accelerating voltage was 5 kV. FTIR was performed using a Transon 27 instrument (Bruker, Germany) equipped with a diamond tip. The solid-state properties of the synthesized amorphous polymer were detected with a Bruker AXS D8 Advance diffractometer with a reflection of θ geometry, using Cu Kα radiation (λ = 1.5418 Å) at a voltage of 40 kV and a current of 30 mA as a radiation source, a receiving slit of 0.2 mm (width), and a high-resolution energy-dispersive detector. The MAS NMR experiment was conducted on a Bruker Advance III 5000 MHz narrow-bore spectrometer via a 4 mm double-resonance MAS probe. ^13^C CPMAS NMR (magic-angle spinning nuclear magnetic resonance) spectra was performed at a frequency of 8 kHz with 2.5 µs ^1^H-shaped 90° pulses for a 2 ms mixing time with a recycle delay of 3 s between acquisitions. NMR spectra were acquired using a Bruker 5000 Ultra Shield spectrometer with a 400 MHz NMR spectrometer (Bruker, Billerica, MA, USA). The dried sample used was ~8 mg under a dynamic nitrogen atmosphere of 100 mL/min, at a temperature range of 30–900 °C and a heating rate of 10 °C/min. High-resolution TEM (HRTEM) analysis was conducted on a JEOL 2100 microscope, operated at 200 kV. Dynamic light scattering (DLS) measurements of the PDA–N@CDs composite and the PDA were performed on a ZetaSizer Nano ZS (Malvern Instruments Ltd., Worcestershire, UK). The samples were prepared by dispersing 2 mg of the PDA–N@CDs composite and the PDA in 10 mL of deionized water and sonicating for 8 min at 25 °C.

### 2.4. Bacterial Culturing and Growth Condition

MRSA ATCC 43,300 was grown overnight at 37 °C under agitation (250 rpm) in a Luria Bertani (LB, Difco) growth medium.

### 2.5. Antibacterial Activity of the PDA–N@CD Composite

The stock solutions of the PDA–N@CD composite and the N@CDs were diluted in an LB medium by two-fold serial dilutions ranging from 0.5 and 0.2 mg/mL for the PDA–N@CD composite and the N@CDs, respectively. Each tube contained 105 colony-forming units (CFU)/mL of *Staphylococcus aureus* (*S. aureus*) and bacteria treated with DDW served as a negative control. Following 48 h of exposure, 10-fold serial dilutions were carried out, and the bacterial cells were plated on LB agar plates and incubated at 37 °C for 20 h. Cell growth was monitored and determined by viable cell counting and expressed as CFUs. All experiments were conducted in triplicate.

## 3. Results and Discussion

### 3.1. Physical and Chemical Characterizations of the N@CDs

**Figure 1:**
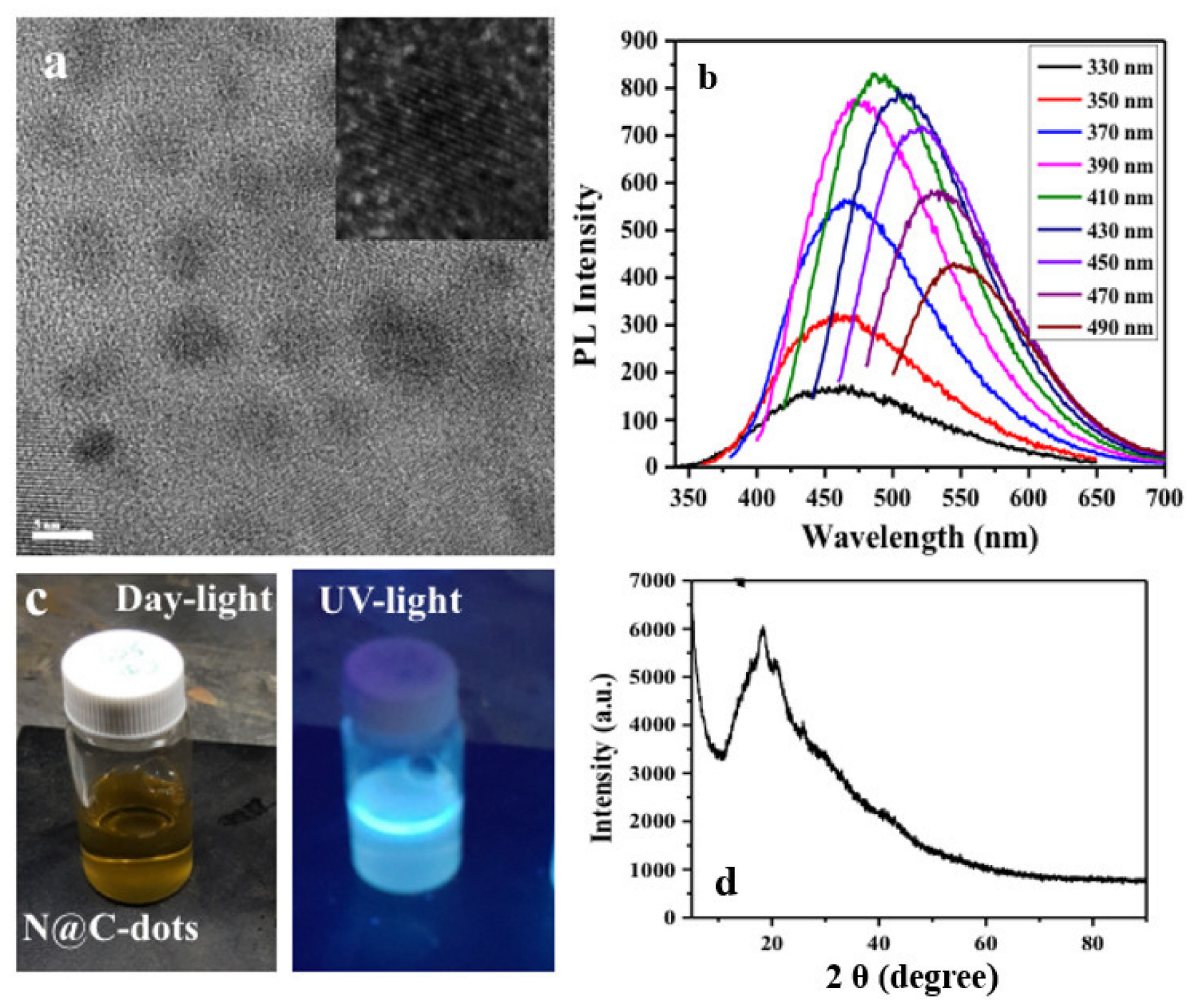
(**a**) HRTEM image of the N@CDs with a d-spacing ~0.215 nm (Insert); (**b**) fluorescence spectra of the N@CDs at different excitation wavelengths; (**c**) colors of optical images of the N@CDs under daylight and UV light; (**d**) XRD of the N@CDs.

The N@CDs (diameter of 4–8 nm and d-spacing of ~0.215 nm; Figure 1a) displayed photoluminescence at excitation wavelengths from 330 to 490 nm, with emission detected from 420 to 590 nm (Figure 1b). A pale-yellow color (Figure 1c) was observed for the synthesized N@CDs with high crystallinity (Figure 1d) under daylight but appeared bluish-white under UV light (365 nm).

### 3.2. Characterization of Polydopamine

**Figure 2:**
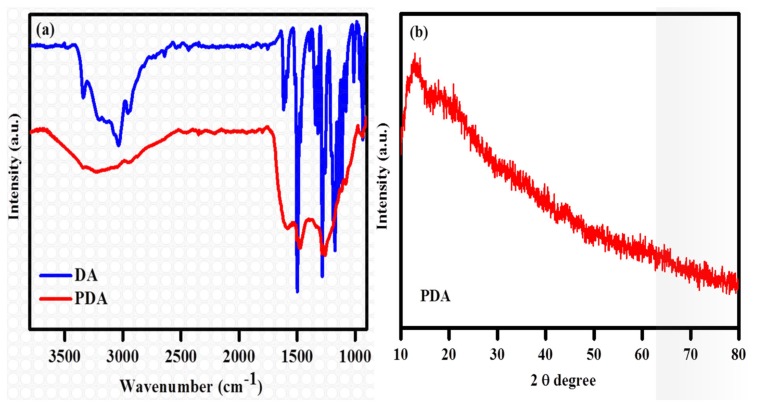
(**a**) FTIR spectra of the DA and the PDA; (**b**) XRD spectra of the PDA.

The FTIR spectrum of the DA exhibits several sharp peaks (Figure 2a), which are in agreement with the literature [41]. In contrast, the FTIR spectrum of the PDA only exhibits three discernible broad peaks, confirming the formation of PDA by the N@CDs. The FTIR spectrum of the PDA has one typical broad peak spanning from 3200 to 3500 cm^−1^, indicating the presence of the hydroxyl moiety of the PDA. The peaks of ~1605 and 1502 cm^−1^ in the FTIR spectrum of the PDA correspond to the indoline and indole structures, respectively. The formation of PDA results in the disappearance of the narrow peaks of the DA molecule between 3000 and 3350 cm^−1^. Indeed, this feature is replaced by the stretching vibrations of the aromatic ring at 1512 cm^−1^ and those of N–H and O–H at 3360 cm^−1^. The peak of ~1468 cm^−1^ is attributed to the shearing vibration of N–H, whereas the peak at 1290 cm^−1^ is the stretching vibration of phenolic C–O.

The XRD pattern of the PDA shows in Figure 2b. The XRD pattern displays two broad peaks at 2θ of ~13.7° and 18.3°–29.3°, evincing the formation of a repeated unit of the DA ring and indicating the polymer chain is highly bonded. These broad peaks indicate the formation of PDA is of an amorphous nature because highly crystalline polymers would be expected to give much sharper peaks.

### 3.3. Particle Size Analysis by Dynamic Light Scattering

**Figure 3:**
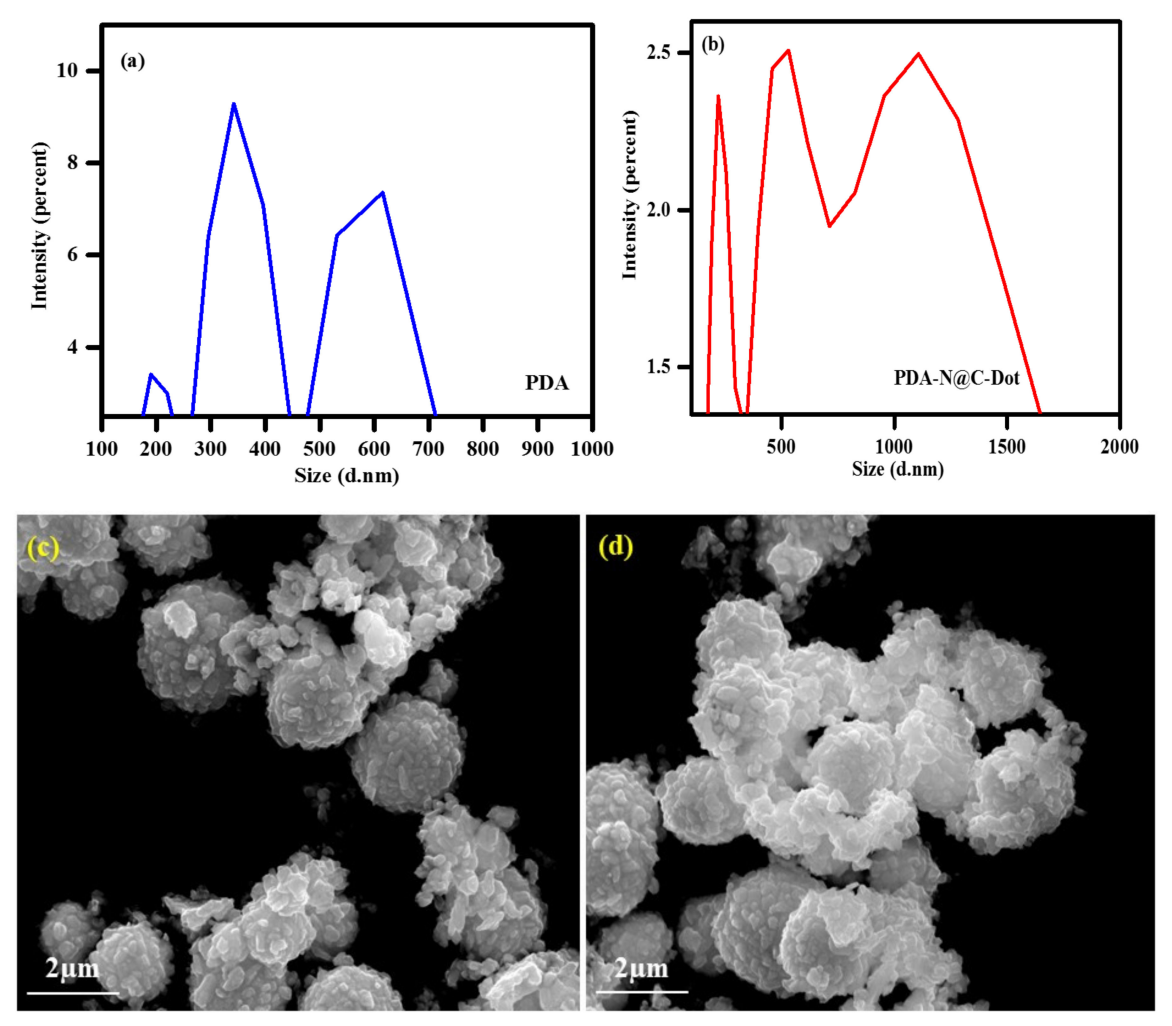
(**a**) designated dynamic light scattering (DLS) measurements of the PDA; (**b**) he PDA–N@CD composite material; SEM images of the PDA (**c**); (**d**) the PDA–N@CD composite material

The PDA was prepared by dispersing 3 mg of the polymer in 10 mL of DDW with bath sonication for 15 min at room temperature. This measurement enabled calculations of the polydispersity indices and the average particle size. The DLS curve of the PDA–N@CD polymer solution reveals the existence of three particle populations (Figure 3b). About 20% of the particle population had a diameter below 300 nm, 40% had nanosized diameters ranging from 300 to 800 nm, and 40% had nanosized diameters ranging from 800 to 1700 nm. The particle sizes of the PDA ranged from 200 to 700 nm in diameter (Figure 3a). Apparently, the PDA was well dispersed in water due to sonication. Most particles in the colloidal material of the PDA–N@CD composite material were composed of microscale particles. The DLS measurement of the PDA (Figure 3a) underwent mild sonication, yielding smaller particles and leading to the disintegration of large polymer particles. The polydispersity indices of the PDA and the PDA–N@CD composite are below 1.0, evidencing the particulate nature of the PDA and the PDA–N@CD composite and strongly indicating good processability of the polymer dispersions. Morphology studies were carried out for the PDA polymer and the PDA–N@CD composite material by scanning electron microscopy (SEM). Figure 3c,d show the SEM images of the PDA and the PDA–N@CD composite material. The SEM images exhibit agglomerated particles with a spherical shape and a broad range of particle sizes ~2 µm. A negative surface charge was observed for both the PDA (−36 mV) and the PDA–N@CD composite material (−39 mV), confirming the stability of these two colloidal solutions.

### 3.4. Solid-State 13C NMR Spectra

**Figure 4:**
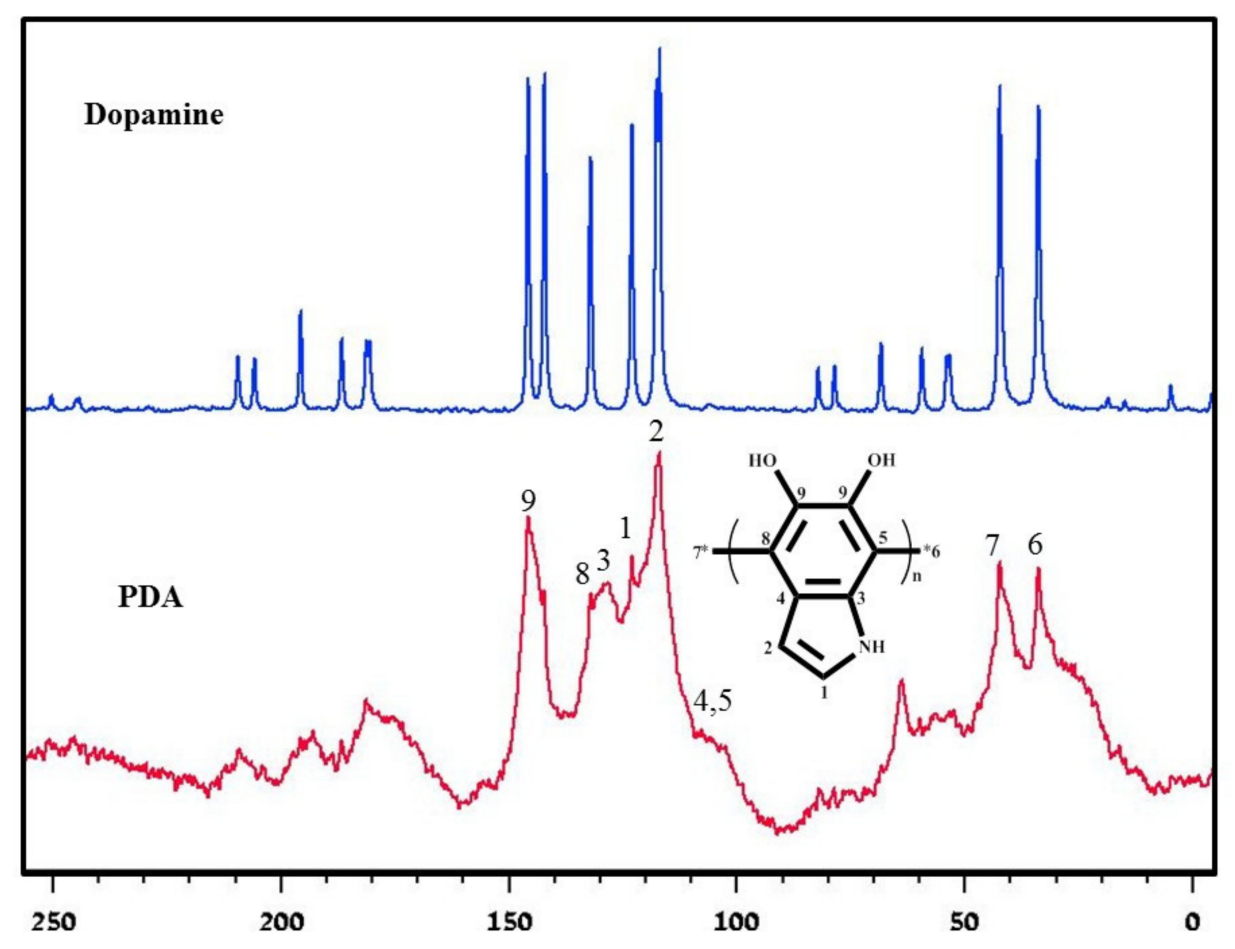
Solid-state ^13^C NMR spectra of the DA and the as-synthesized PDA (The unit of X-axis: ppm).

The PDA polymer product was further characterized by solid-state ^13^C NMR measurements, which were conducted in a cross-polarization magic-angle spinning mode. The solid-state ^13^C NMR spectrum exhibits many DA peaks as well as broad peaks due to the formation of large molecules with slow dropping times relative to the timescale of NMR relaxation (Figure 4). In particular, The solid-state ^13^C NMR spectrum of the as-synthesized PDA displays two broad peaks spanning from 100 to 150 ppm and from 20 to 50 ppm, respectively, for aromatic carbons. All the DA single-molecule sharp peaks disappear, and the ^13^C NMR spectrum of the PDA only displays a few broad peaks, indicating the polymerization of DA to form PDA. The spectral features could be attributed to the aromatic carbons of the PDA. The solid-state NMR spectrum of the DA displays the peaks of the C-7 and C-8 carbons at 33 and 41 ppm, respectively, corresponding to unprotonated aromatic carbons. The shoulder peaks around 105 and 111 ppm could be attributed to the C-4 and C-5 carbons, respectively, and the peaks at 115 and 121 ppm could be attributed to the protonated C-1 and C-2 carbons, respectively. The aromatic carbons (C-9) bound the hydroxyl groups, which appeared at around 149 ppm.

### 3.5. Antibacterial Activity

**Figure 5:**
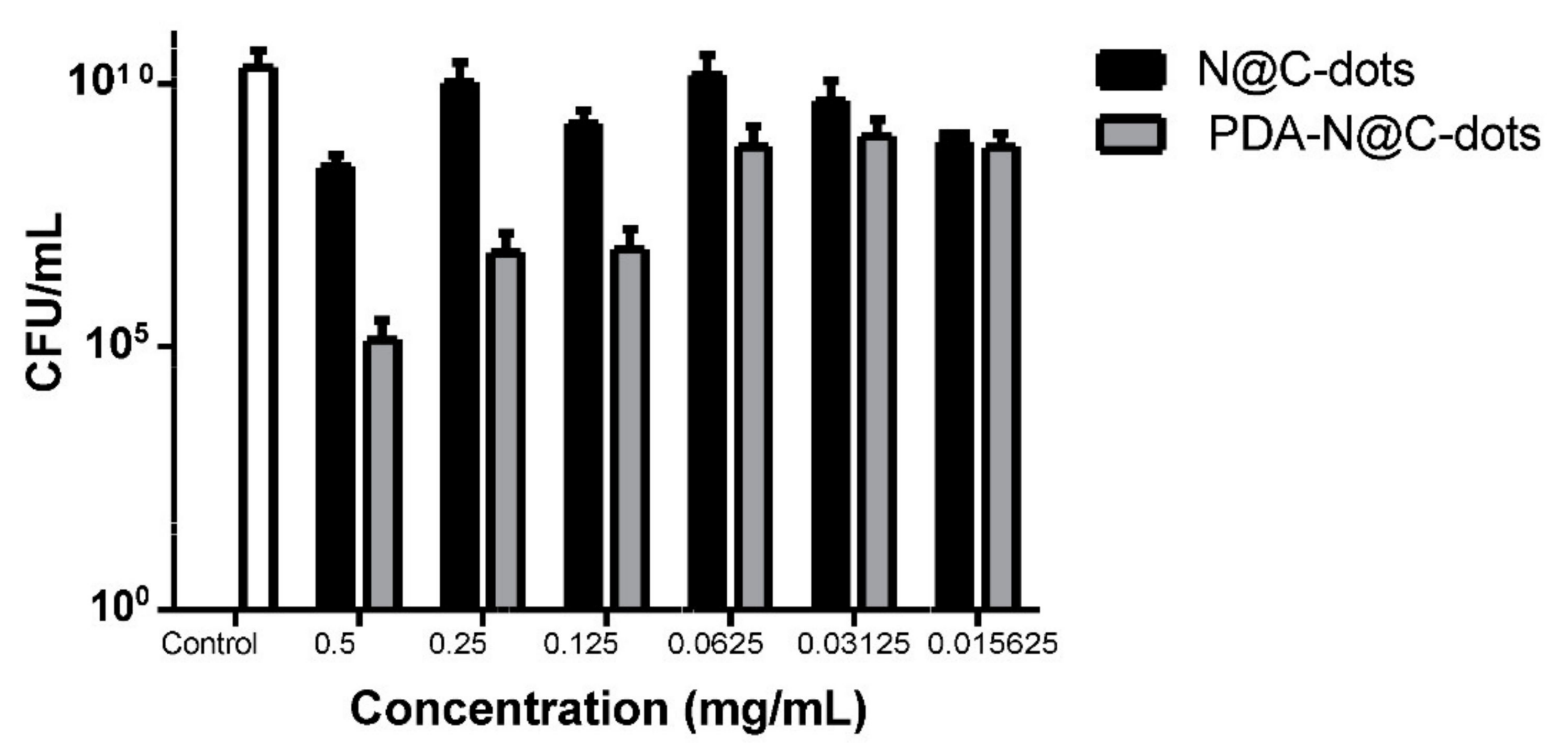
Antibacterial activity of the PDA–N@CD composite material against the MRSA following 48 h exposure. The results are presented as the average of three independent experiments ± SD.

The PDA–N@CD composite material reduced the CFU of the MRSA bacteria by 3.5 logs following exposure to 0.5 mg/mL of the PDA–N@CD composite material (Figure 5). Importantly, a lower concentration of the material, 0.125 mg/mL, still demonstrated a significant effect on the viability of MRSA (Figure 5).

PDA has been shown to exert antimicrobial activity, triggered by charged functional groups in a polymer [43,44]. These charged groups in the polymer backbone and their potential electrostatic and ionic interactions react with a bacterial cell wall to compromise its integrity, leading to eventual cell lysis. The charges present in the PDA are generated by the protonation of amino groups in an acidic condition or can be introduced through structural modification. PDA is obtained by the cyclic addition during the polymerization reaction caused by CDs with a higher polymeric charge density. Since the charges are stable in the polymer, they probably influence antimicrobial activity. Catechol groups of PDA have been known to form strong reversible noncovalent and irreversible covalent interactions with organic chemicals of bacterial surfaces, which include hydrogen bonding and π–π electron interaction with another aromatic ring [45]. The PDA polymer with high adhesion property binds a bacterial membrane and eventually coats a cell surface to restrict the diffusion of nutrients and wastes in and out of the cytosol, i.e., restricting the cell growth and replication. Eventually, the bacterium would be completely embedded in PDA with a distorted shape. This behavior was observed for *Escherichia coli* [46]. This work unraveled a considerable antibacterial effect against *S. aureus* (Gram-positive), and a similar mechanism could be suggested for cell death. The binding of a bacterial membrane with PDA plays an important role in the death of this pathogen, which has been known to release different cytolytic toxins [47,48] to damage the host tissue. In addition, *S. aureus* releases ~20 enterotoxins and enterotoxin-like toxins to cause emesis and diarrhea. Apparently, PDA is also capable of binding such toxins to circumvent their detrimental effect on the infected cell. This generic combined mechanism should be extended to other antibiotic-resistant bacteria, regardless of whether they are Gram-negative or Gram-positive. The antimicrobial property of PDA could also be attributed to the presence of catechol, which autoxidizes in the presence of oxygen to form semiquinone and quinone [42]. During this oxidation process at neutral pH and room temperature [42], ROS are generated, including superoxide anions (O^2–^) and hydrogen peroxide-two well-known disinfectants with antimicrobial activity against both Gram-negative and Gram-positive bacteria.

## 4. Conclusions

In brief, this study reports on the colloidal dispersion of a PDA–N@CD composite material. We present a simple one-step, high-speed dissolving method of combining a polymer with N@CD. The obtained polymer presented good antibacterial activity against MRSA, a widespread bacterium and a major cause of nosocomial infections. The colloidal dispersion of the PDA–N@CD composite material at a low concentration was still capable of eradicating the MRSA without other added metal or metal oxide nanoparticles. The novelty of the current work lies in the simple synthesis of PDA with N@CDs as the only initiator, which is a green approach. Microorganisms can be entrapped by PDA to interfere with microbial growth and mitosis. The generation of ROS is another possible mechanism of cell death, which is applied to both Gram-positive and Gram-negative bacteria.
